# Supplementary material for: Inhibition of ATM Increases the Radiosensitivity of Uveal Melanoma Cells to Photons and Protons
Source: Cancers (Basel). 2020 May 28;12(6):1388. doi: 10.3390/cancers12061388 (PMC7352388; doi:10.3390/cancers12061388)
Supplement: Supplementary file 1 [file cancers-12-01388-s001.pdf]

cancers

Supplementary Materials

Inhibition of ATM Increases the Radiosensitivity of Uveal Melanoma Cells to Photons and Protons

Rumana N. Hussain, Sarah E. Coupland, Jakub Khzouz, Helen Kalirai and Jason L. Parsons

Table S1. ATM staining (% positive tumor cells) in proton-irradiated and primary enucleation UM samples.

	**Proton-irradiated (*n* = 32)**	**Non-irradiated (*n* = 20)**	**Statistical Analysis**
ATM	29.69%	31.25%	$p = 0.911$

Table S2. ATM staining (% positive tumor cells) in proton-irradiated UM samples and association with tumor recurrence.

	**Tumor Recurrence/Growth (*n* = 18)**	**Neovascular Complications (*n* = 14)**	**Statistical Analysis**
ATM	32.78%	25.71%	$p = 0.326$

Table S3. Correlative analysis of ATM staining (% positive tumor cells) and high-risk UM tumor characteristics.

		**ATM Staining (%) (n=20)**	**Statistical Analysis**
Chromosome 3 status	Disomy 3	27.14	$p = 0.516$
	Monosomy 3	35.00	
Cause of enucleation	Radiation complications	25.71	$p = 0.498$
	Tumor related	32.78	
Ciliary body structure	No	27.33	$p = 0.670$
	Yes	31.76	
Tumor size	Small (<15 mm)	30.00	$p = 0.917$
	Large (>15 mm)	28.75	
Lymphocytic infiltration	No	80.00	$p = 0.740$
	Yes	28.06	
Macrophages	Yes	All	
Plasma cells	No	29.17	$p = 0.909$
	Yes	30.36	
Mitotic count			$p = 0.625$
Time to enucleation			$p = 0.719$

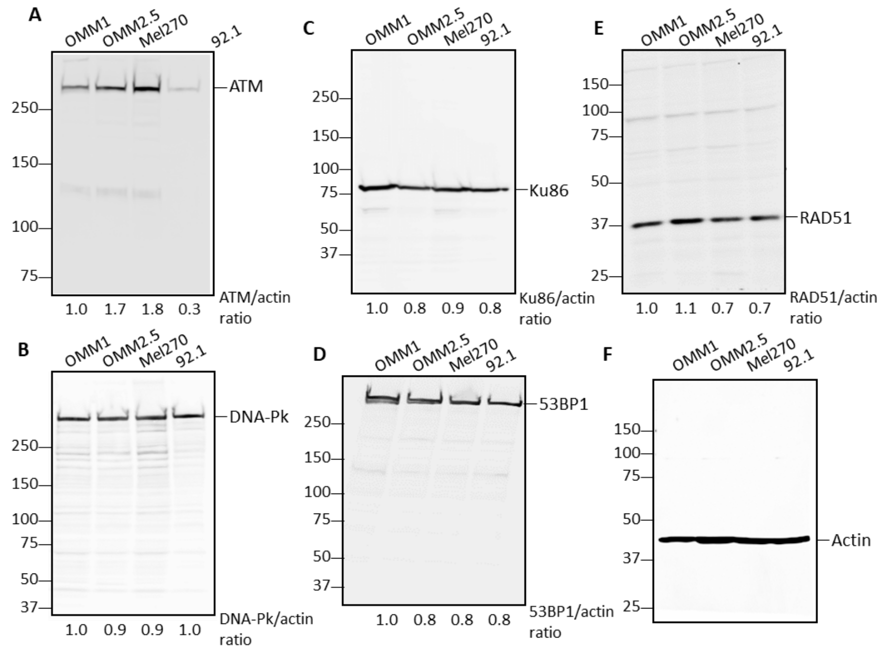

Figure S1. Expression of DSB repair proteins in UM cell lines. Whole cell extracts from UM cell lines were prepared and analysed by immunoblotting with either (A) ATM or (B) DNA-Pk, (C) Ku86, (D) 53BP1, (E) RAD51 or (F) actin antibodies. Representative images are shown, along with the relative protein ratio to actin, normalised to those in the OMM1 cells which was set to 1.0.

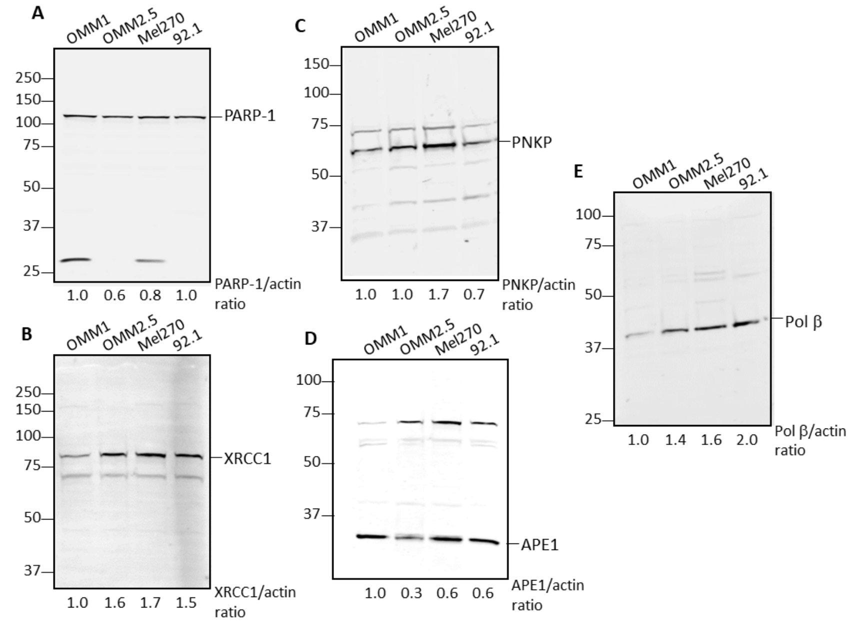

Figure S2. Expression of BER and SSB repair proteins in UM cell lines. Whole cell extracts from UM cell lines were prepared and analysed by immunoblotting with either (A) PARP-1, (B) XRCC1, (C) PNKP, (D) APE1 or (E) Pol β antibodies. Representative images are shown, along with the relative protein ratio to actin, normalised to those in the OMM1 cells which was set to 1.0.

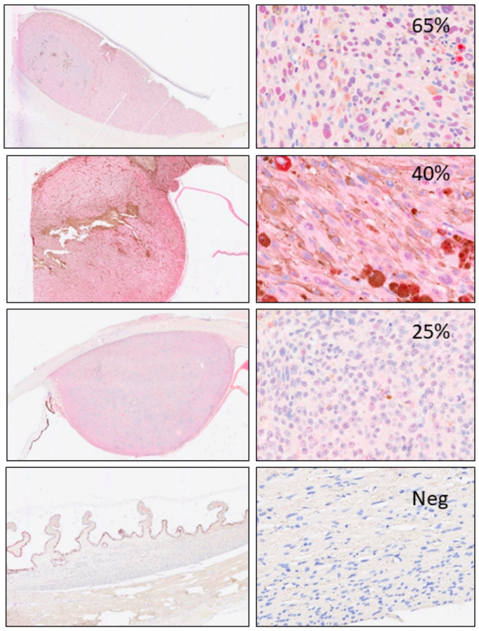

Figure S3. Variability in the staining of ATM observed in cells from UM tissues. Immunohistochemical staining using ATM antibodies and detection was performed, along with counter-staining using Mayer's hematoxylin. Stained cells were evaluated using both a x10 (left panels) and a x40 objective (right panels), and shown are examples of tissues evaluated as either negative staining for ATM (Neg), or with increased staining (25–40%) ATM staining.

© 2020 by the authors. Licensee MDPI, Basel, Switzerland. This article is an open access article distributed under the terms and conditions of the Creative Commons Attribution (CC BY) license (http://creativecommons.org/licenses/by/4.0/).
